# Horner syndrome as a postoperative complication of thyroid surgery: a systematic review

**DOI:** 10.3389/fendo.2025.1664870

**Published:** 2025-10-01

**Authors:** Tianhao Xie, Yan Fu, Xiaoshi Jin, Qingxu Meng, Yang Liu, Xiaoshuang Liu, Zheng Niu, Xinli Sun, Lingyun Liu

**Affiliations:** ^1^ Department of General Surgery, Affiliated Hospital of Hebei University, Baoding, Hebei, China; ^2^ Basic Research Key Laboratory of General Surgery for Digital Medicine, Affiliated Hospital of Hebei University, Baoding, Hebei, China; ^3^ Department of Ophthalmology, Baoding No.1 Central Hospital, Baoding, Hebei, China

**Keywords:** thyroid, Horner syndrome, complication, thyroidectomy, thyroid cancer

## Abstract

**Objective:**

This systematic review aims to enhance surgeons’ awareness of Horner Syndrome (HS) as a rare yet significant complication following thyroid surgery (TS).

**Data sources:**

Web of Science, PubMed, Cochrane Library, and Embase.

**Review methods:**

Based on the PRISMA framework, a comprehensive literature search was conducted covering the period from January 1, 2000, to June 1, 2025.

**Results:**

Out of the 308 articles retrieved, 50 were included in this review, comprising 14 case series and 36 case reports. These studies documented a total of 40 cases of HS following TS. The overall incidence of HS was found to be 0.25%, with a higher incidence in open surgery (0.41%) compared to endoscopic surgery (0.15%). The incidence rate among children undergoing open surgery was 1.84%, which was higher than that among adults (0.22%). Malignant cases accounted for 67.5%, while benign cases represented the remaining 32.5%. HS symptoms typically manifested within 3 days post-surgery, with ptosis being the most common presentation. It rarely affects ocular function but may lead to decreased vision or heterochromia. Short-term steroid and neurotrophic therapy demonstrated some efficacy in alleviating symptoms, and complete recovery was more likely to occur within one year.

**Conclusion:**

HS represents a rare yet significant complication of TS, primarily attributed to surgical trauma to the cervical sympathetic chain (CSC). Clinicians must remain vigilant regarding this complication and employ meticulous surgical techniques to prevent CSC injury.

## 1 Background

Horner Syndrome (HS), first described in 1869 by Swiss ophthalmologist Johann Friedrich Horner (1), is a clinical condition resulting from paralysis of the oculosympathetic pathway (OSP). Its characteristic triad of symptoms includes ptosis, miosis, and anhidrosis. Primary etiologies of HS encompass head and neck tumors, trauma, brainstem hemorrhage, infarction, myelitis, carotid artery dissection, infections, iatrogenic causes (e.g., surgical procedures), and other factors (2).

As early as 1915, Kaelin (3) reported two cases of HS following thyroid surgery (TS), which were documented but not published by Kappeler between 1865 and 1870. Both patients developed postoperative wound infections, leading Kaelin to hypothesize that infection irritated the cervical sympathetic nerve, resulting in HS. In 1965, Smith (4) documented seven additional cases of HS after TS; notably, these patients had no complications, including infection or hematoma. Based on these findings, Smith proposed that HS primarily resulted from surgical damage to the cervical sympathetic chain (CSC).

However, for over two decades, no further reports of HS as a postoperative complication of TS were published. Not until 1990, when Buhr (5) described three cases of HS following medullary thyroid carcinoma surgery, did this complication begin to garner attention in the surgical community. In the past decade, the number of reports of HS after TS has increased significantly. This article presents a case of HS after TS and provides a comprehensive analysis of its incidence, histopathology of associated tumors, surgical techniques and extent of resection, clinical manifestations, onset time, and recovery time, based on a review of literature published from 2000 to 2025. The aim of this study is to enhance surgeons’ awareness of this potential complication.

## 2 Materials and methods

### 2.1 Case report

**Figure 1:**
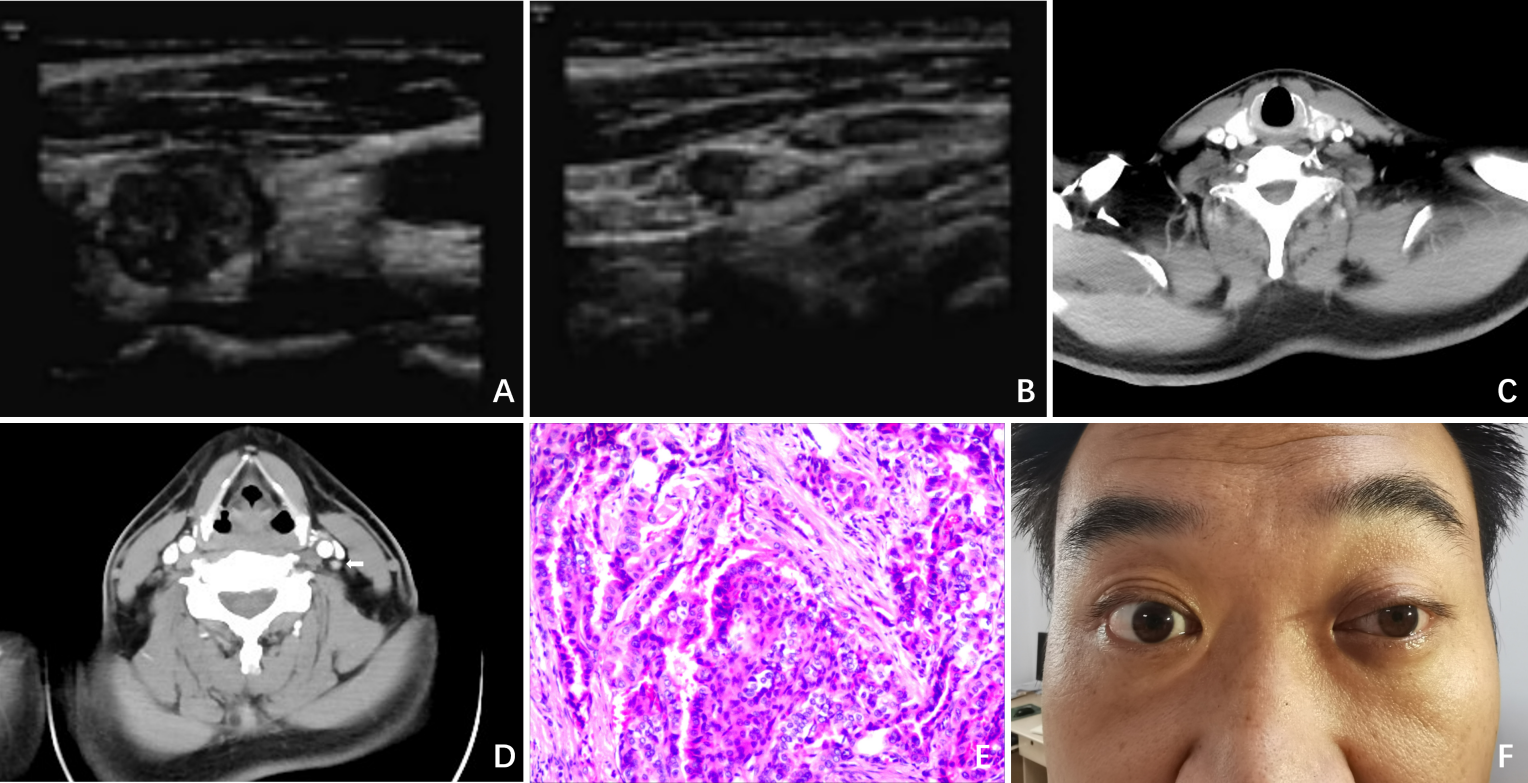
Clinical data. **(A)** Ultrasound revealed a nodule situated in the left lobe of the thyroid, measuring 1.4cm x 1.4cm x 1.6cm, with low echogenicity and containing strong echo spots; **(B)** Ultrasound revealed a 0.6cm lymph node containing strong echo spots in the vicinity of the left carotid artery sheath; **(C)** Enhanced CT revealed a significantly enhanced nodule in the left lobe of the thyroid; **(D)** Enhanced CT revealed a markedly enhanced lymph node at level II of the left neck (white arrow); **(E)** Postoperative pathology indicated PTC; **(F)** On POD 3, ptosis of the left eyelid, miosis, and anhidrosis on the ipsilateral face was observed in the patient.

A 36 - year - old male underwent an ultrasound examination, during which a thyroid nodule was detected. The ultrasound revealed a nodule situated in the left lobe of the thyroid, measuring 1.4 cm x 1.4 cm x 1.6 cm, with low echogenicity and containing strong echo spots. The nodule was classified as TI - RADS 4a. Additionally, a 0.6 cm lymph node, also containing strong echo spots, was identified in the vicinity of level III of the left carotid artery sheath (**Figures 1A, B**). The patient denied having any clinical symptoms, a personal history, or a family history of thyroid disease. Upon physical examination, a firm nodule with a diameter of 2 cm was palpable in the left thyroid lobe. An enhanced computed tomography (CT) scan revealed a significantly enhanced nodule in the left lobe of the thyroid, along with a markedly enhanced lymph node at level III of the left neck, which was consistent with the lymph node findings assessed by ultrasound (**Figures 1C, D**). Subsequent fine-needle aspiration (FNA) biopsies of both the thyroid nodule and the lymph node confirmed the presence of papillary thyroid carcinoma (PTC) cells.

The patient then underwent a total thyroidectomy (TT), accompanied by bilateral central lymph node dissection (CLND) and lateral lymph node dissection (LLND) that encompassed left levels II, III, IV, and V. Throughout the entire surgical procedure, we used an ultrasonic scalpel to incise the tissues while avoiding exposure of the CSC. Meanwhile, intraoperative nerve monitoring (IONM) was employed to ensure the normal transmission of signals from the recurrent laryngeal nerve and vagus nerve. Stimulation of the phrenic nerve and accessory nerve resulted in the expected muscle movements, confirming their functionality. The operation proceeded smoothly without any complications or unexpected findings. Postoperative pathological examination further confirmed the diagnosis of PTC (**Figure 1E**). The status of lymph node metastasis is as follows: right level VI (0/4), left level II (0/4), level III (3/18), level IV (2/9), level V (0/2), and level VI (4/6).

On postoperative day (POD) 3, we observed ptosis of the left eyelid, miosis, and anhidrosis on the ipsilateral face, with no signs of vascular dilatation (**Figure 1F**). Consequently, a thorough ocular examination was conducted collaboratively by a neurologist and an ophthalmologist. After excluding other potential complications such as hematoma, dyspnea, inflammation, or vocal cord issues, the patient was diagnosed with HS.

After obtaining the patient’s consent, a treatment plan was initiated, consisting of a 3 - day intravenous dexamethasone course (10 mg once daily) and a 6 - day intravenous mecobalamin course (0.5 mg every 2 days). Following the administration of glucocorticoids and neurotrophic drugs, there was slight improvement in ptosis and miosis. However, during a follow - up visit 2 years after the surgery, the symptoms had not completely resolved, and the patient was only taking levothyroxine sodium without any other medication.

### 2.2 Method of literature search

This systematic literature review follows the guidelines recommended by PRISMA (Preferred Reporting Items for Systematic Reviews and Meta-Analyses). The electronic databases Pubmed, Embase, Cochrane Library, and Web of Science were searched from January 1, 2000, to June 1, 2025. A combination of MeSH terms (“Horner syndrome” [MeSH Terms], “Thyroid” [MeSH Terms]) and free-text words were utilized to search (“Horner syndrome”; “thyroid surgery”; “complication”; “thyroidectomy”).

### 2.3 Inclusion and excluding criteria

This study encompassed articles that reported cases of HS resulting from TS for thyroid diseases. Articles related to animal experiments, academic theses, conference proceedings, reviews, meta-analyses, as well as cases involving interventional measures such as ablation and FNA, were excluded from this study.

Inclusion Criteria: (1) Thyroid surgery can be performed via an open or endoscopic approach. (2) Documented occurrence of HS as a post - TS complication.

Exclusion Criteria: (1) Non-primary research literature, encompassing dissertations/theses, conference abstracts, review articles, and meta - analyses. (2) Cases with pre-existing HS prior to the initiation of TS. (3) Studies that fail to report outcomes related to HS. (4) Animal-based studies or *in vitro* experimental investigations. (5) Duplicate publications or studies for which the full texts are inaccessible. (6) Articles related to interventional measures such as ablation therapy and FNA.

### 2.4 Article selection process

**Figure 2:**
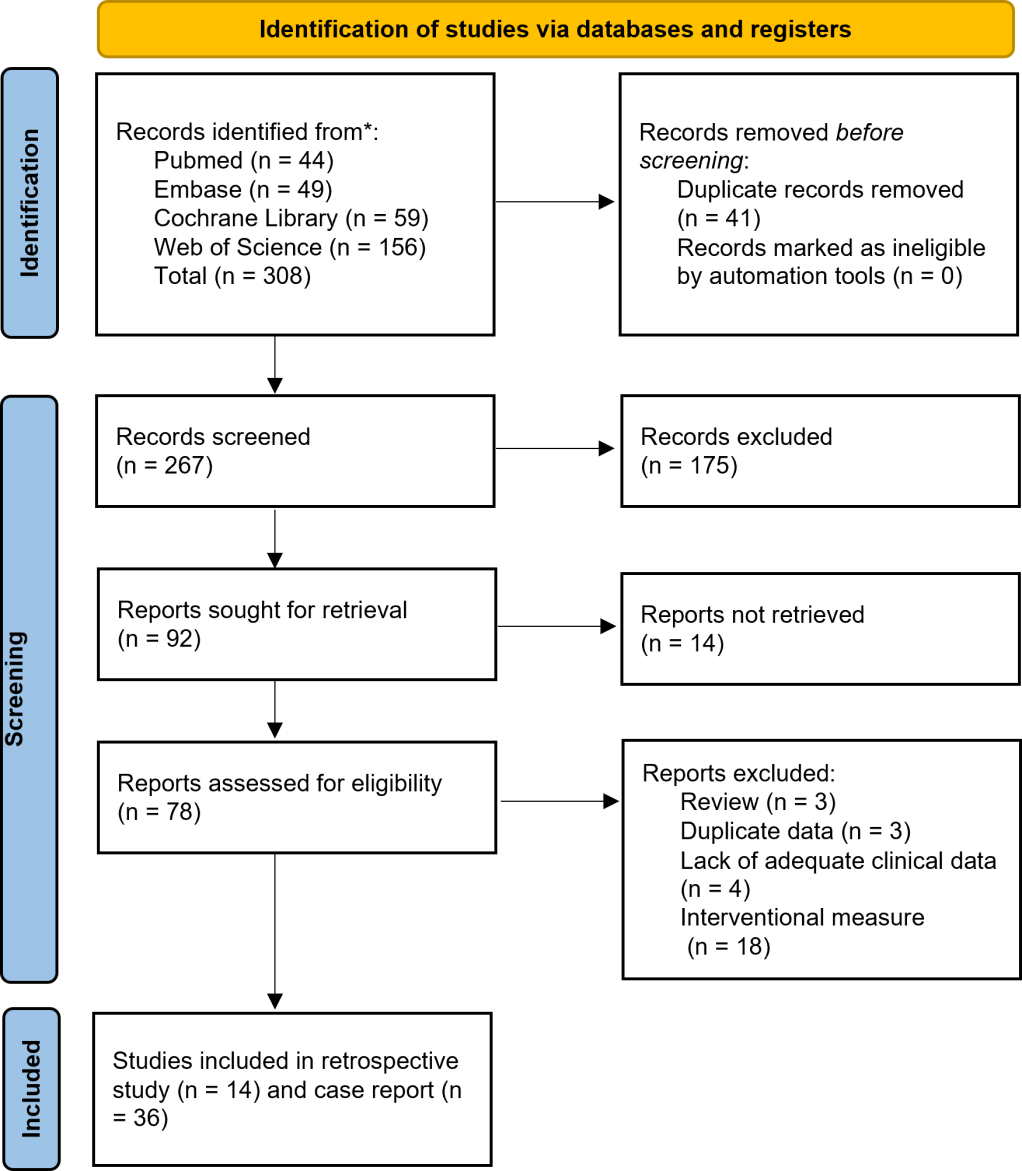
Preferred Reporting Items for Systematic Reviews and Meta-analyses^24^ diagram resembling electronic database search and inclusion/exclusion process of the review. *Date of last search June 1, 2025.

Retrieved citations were organized, and duplicates were removed using EndNote software (Clarivate Analytics). The study selection process was independently conducted by two researchers. Initial screening involved reviewing titles and abstracts against predefined inclusion and exclusion criteria. Full-text articles of potentially eligible studies were then assessed for final eligibility. Following independent evaluations, researchers cross-checked their selection results. Any discrepancies were resolved through consultation with a senior investigator to achieve consensus. A detailed flowchart of the selection process is provided in **Figure 2**.

### 2.5 Data extraction and analysis

The study records encompassed a range of details, including the incidence of HS as a complication of TS, histopathology of tumors, surgical approaches employed, extent of resection, clinical manifestations, onset time of HS, other associated complications, specific treatment administered for HS, follow-up duration, and recovery outcomes. Surgical procedures are classified into two major categories: open surgery (including conventional and minimally invasive approaches or mini-incision thyroidectomy), endoscopic surgery (including endoscopic thyroid surgery (EST), robotic-assisted endoscopic thyroidectomy (RAET), and video-assisted thyroidectomy (VAT)). All these data points were systematically organized and presented in tables. SPSS software (version 22.0) was used to calculate the 95% confidence interval (CI) using the Wilson Score Interval Method.

### 2.6 Quality assessment

The Joanna Briggs Institute (JBI) critical appraisal tools were employed to evaluate the quality of the case reports and series. This independent assessment was carried out by three authors. For the generation of risk of bias graphs and summaries pertaining to both case reports and case series, the Review Manager (RevMan^®^) software (Version 5.4), published by The Cochrane Collaboration, was utilized. During the assessment process, if a study achieved a ‘yes’ response rate of 70% or higher to the appraisal questions, its risk of bias was deemed low; if the ‘yes’ response rate ranged from 50% to 69%, the risk of bias was considered moderate; and if the ‘yes’ response rate did not exceed 49%, the risk of bias was classified as high.

## 3 Results

Of the 308 articles retrieved, 50 were selected for inclusion in this study, comprising 14 case series and 36 case reports. Among these, 18 articles (including 14 case series and 4 case reports) provided data on the incidence of HS (6–23) (**Table 1**). Additionally, a total of 40 cases (including one from our own institution) were documented with detailed records in 36 case reports (7, 18, 20, 24–56) (**Table 2**). No cases of transoral endoscopic thyroidectomy vestibular approach (TOETVA) were retrieved in the search.

**Table 1:** Incidence of Horner syndrome related to thyroid surgery.

Author (year)	Approach	Total cases	HS cases	Incidence rate(%)	95% confidence interval
Open surgery	——	4133	17	0.41	0.22%, 0.61%
van Santen HM (2004) (6)	Conventional	25(children)	2	8.00	0.80%, 15.20%
Cozzaglio L (2008) (7)	Conventional	495	1	0.20	0.02%, 0.78%
Tan C (2009) (8)	Minimally invasive	86	1	1.16	0.01%, 4.43%
Lee YS(2010) (9)	Conventional	2636	5	0.19	0.11%, 0.30%
Karnak I (2011) (10)	Conventional	16(children)	1	6.25	0.06%, 18.58%
Welch K (2013) (11)	Conventional	45	2	4.44	0.22%, 9.22%
Kundel A (2014) (12)	Conventional	177(children)	1	0.56	0.06%, 1.69%
Al-Hakami HA (2019) (13)	Conventional	456	1	0.22	0.02%, 0.67%
Zhang Y (2022) (14)	Conventional	97	2	2.06	0.21%, 4.04%
Hassan I (2023) (15)	Minimally invasive	100	1	1.00	0.01%, 3.69%
Endoscopic surgery	——	6564	10	0.15	0.01%, 0.15%
Lee J (2011) (16)	RAET	1043	1	0.10	0.001%, 0.37%
Kang SW (2011) (17)	RAET	1000	1	0.10	0.001%, 0.37%
Ying X (2013) (18)	VAT	537	2	0.37	0.04%, 1.40%
Ban EJ (2014) (19)	RAET	3000	1	0.03	0.0003%, 0.20%
Meng K (2015) (20)	ETS	416	2	0.48	0.05%, 1.44%
Nakajo A (2017) (21)	ETS	16	1	6.25	0.62%, 36.94%
Ge JN (2023) (22)	ETS	521	1	0.19	0.02%, 0.72%
Zhang DG (2023) (23)	ETS	31	1	3.23	0.32%, 9.68%
Total	——	10697	27	0.25	0.16%, 0.35%

RAET, robotic-assisted endoscopic thyroidectomy; VAT, video-assisted thyroidectomy; ETS, endoscopic thyroid surgery.

**Table 2:** Reported cases of Horner syndrome related to thyroid surgery.

Author (year)	Histopathology	Approach	Extent of operation	Time of symptom onset	Symptoms	Other complications	Special treatment	Follow-up time	Recovery outcome
Cozzaglio L (2008) (7)	Basedow–Graves’ disease	Conventional	TT	POD 2	Ptosis, miosis, enophthalmos	None	NM	4 days	Resolved
de Silva (2010) (24)	Degenerating colloid nodule	Conventional	UT	POD 7	Ptosis, miosis, anhidrosis	None	NM	3 months	Incomplete resolved
Italiano D (2011) (25)	NG	Conventional	TT	SD	Ptosis, miosis, enophthalmos	Vocal cord paralysis	NM	2 months	Incomplete resolved
Meng LW (2011) (26)	PTC	Conventional	UT+ CLND	POD 2	Ptosis	None	Dexamethasone, mecobalamin	1 week	Resolved
	PTC	Conventional	UT+ CLND	POD 1	Ptosis, miosis	None	Dexamethasone, mecobalamin	3 months	Resolved
Vilallonga R (2012) (27)	ATC	Conventional	UT (reoperation)	POD 1	Ptosis, miosis	Vocal cord paralysis	NM	2 years	No improvement
González-Aguado R (2012) (28)	PTC	Conventional	TT+ CLND+ LLND	SD	Ptosis, miosis, anhidrosis	NM	NM	5 months	Incomplete resolved
Ying X (2013) (18)	PTC	VAT	UT+ CLND	POD 2	Ptosis, miosis	None	Steroid	4 months	Incomplete resolved
	PTC	VAT	UT+ CLND	POD 3	Ptosis, miosis	None	None	5 days	Resolved
Aslankurt M (2013) (29)	NG	Conventional	ST	SD	Ptosis, miosis, anhidrosis	None	NM	6 months	No improvement
Sandoval MA (2015) (30)	ATC	Conventional	TT+ CLND+ LLND	NM	Ptosis, miosis	NM	NM	NM	NM
Meng K (2015) (20)	PTC	ETS	UT+ CLND	POD 1	Ptosis, miosis	None	Mecobalamin	11 months	Resolved
	PTC	ETS	UT+ CLND	POD 3	Ptosis, miosis	None	None	1 months	Resolved
Ulusoy MO (2016) (31)	Benign	Conventional	TT	NM	Ptosis, miosis, decreased vision, heterochromia	NM	NM	4 years	Incomplete resolved
Giannaccare G (2016) (32)	PTC	Conventional	TT	SD	Ptosis, miosis	None	NM	6 months	No improvement
Mastronikolis NS (2016) (33)	MTC	Conventional	TT+ CLND+ LLND	POD 1	Ptosis, miosis	Vocal cord paralysis	NM	4 months	Resolved
Martínez-Álvarez A (2016) (34)	PTC	Conventional	TT+ CLND+ LLND	POD 3	Ptosis, miosis	Vocal cord paralysis	NM	NM	Resolved
Seneviratne SA (2016) (35)	NG	Conventional	TT	POD 7	Ptosis, miosis, enophthalmos	None	NM	1 year	Incomplete resolved
Lee MS (2016) (36)	Malignancy	Conventional	TT+ CLND+ LLND	POD 2	Ptosis, miosis, anhidrosis	Eye and facial pain	Dexamethasone, analgesic	6 months	Incomplete resolved
Liu YZ (2016) (37)	NG	Conventional	TT+ CLND	POD 3	Ptosis	None	mecobalamin	3 months	Incomplete resolved
Hu X (2017) (38)	PTC	VAT	UT+ CLND + LLND	POD 2	Ptosis, miosis, enophthalmos	None	Dexamethasone, mecobalamin	1 year	Incomplete resolved
Foma W (2017) (39)	Parapharyngeal ectopic goitre	Conventional	UT	POD 4	Ptosis, miosis, enophthalmos	None	NM	1 year	No improvement
Demiral M (2017) (40)	NG	Conventional	TT	POD 2	Ptosis, miosis	None	None	6 months	Resolved
Perréard M (2019) (41)	Benign	Conventional	UT	SD	Ptosis, miosis	None	NM	3 months	No improvement
Sapalidis K (2019) (42)	PTC	Conventional	UT+ CLND + LLND	SD	Ptosis, miosis, enophthalmos	None	NM	2 months	Incomplete resolved
McCrory D (2020) (43)	NG	Conventional	UT	POD 13	Ptosis, miosis, anhidrosis	None	NM	8 months	Incomplete resolved
Janjua MH (2021) (44)	NG	Conventional	TT	POD 2	Ptosis, miosis, anhidrosis, enophthalmos	None	Prednisolone	6 months	Incomplete resolved
Punda A (2021) (45)	PTC	Conventional	LLND (reoperation)	POD 2	Ptosis, miosis, anhidrosis	None	Methylprednisolone	6 months	Incomplete resolved
Min Y (2021) (46)	PTC	ETS	TT+ CLND	POD 3	Ptosis, miosis, anhidrosis	None	Mecobalamin, vitamin B1	3 months	Resolved
Lee SH (2021) (47)	PTC	RAET	TT	SD	Ptosis, miosis	None	None	12 months	No improvement
Palmer EM (2022) (48)	PTC	Conventional	TT+ CLND + LLND	SD	Ptosis, miosis, decreased vision	None	NM	6 weeks	No improvement
Xie T (2022) (49)	PTC	ETS	UT+ CLND	POD 1	Ptosis, miosis, anhidrosis,	None	Dexamethasone, mecobalamin	6 months	Resolved
Deng Y (2023) (50)	PTC	Conventional	UT+ CLND	POD 3	Ptosis, miosis	None	Mecobalamin, vitamin B1	1 week	Resolved
Carsote M (2023) (51)	MTC	Conventional	TT	POD 27	Ptosis, miosis, dry eye, conjunctival discomfort, nasal congestion	Chylous leakage	Aspiration drainage	3 months	Resolved
Arishi AA (2023) (52)	PTC	Conventional	TT+ CLND	SD	Ptosis, miosis, anhidrosis	Vocal cord paralysis	NM	8 months	No improvement
Rauniyar N (2023) (53)	Follicular adenoma	Conventional	UT	POD 1	Ptosis, miosis, enophthalmos, decreased vision	None	None	8 months	Resolved
Manoharan S (2023) (54)	PTC	Conventional	LLND (reoperation)	POD 7	Ptosis, miosis	None	None	NM	Incomplete resolved
Chen H (2024) (55)	PTC	ETS	UT+ CLND	SD	Ptosis, anhidrosis	None	None	6 weeks	Resolved
Tok M (2025) (56)	Malignancy	Conventional	TT+ CLND	SD	Ptosis, miosis, anhidrosis	None	NM	NM	NM
Personal cases	PTC	Conventional	TT+ CLND + LLND	POD 3	Ptosis, miosis, anhidrosis	None	Dexamethasone, mecobalamin	1 years	Incomplete resolved

NG, Nodular goiter; ATC, anaplastic thyroid carcinoma; PTC, papillary thyroid carcinoma; MTC, medullary thyroid carcinoma; VAT, video-assisted thyroidectomy; ETS, endoscopic thyroid surgery; RAET, robotic-assisted endoscopic thyroidectomy; UT, unilateral thyroidectomy; TT, total thyroidectomy; ST, subtotal thyroidectomy; CLND, central lymph node dissection; LLND, lateral lymph node dissection; SD, surgery day; POD, postoperative day; NM, not mentioned.

**Figure 3:**
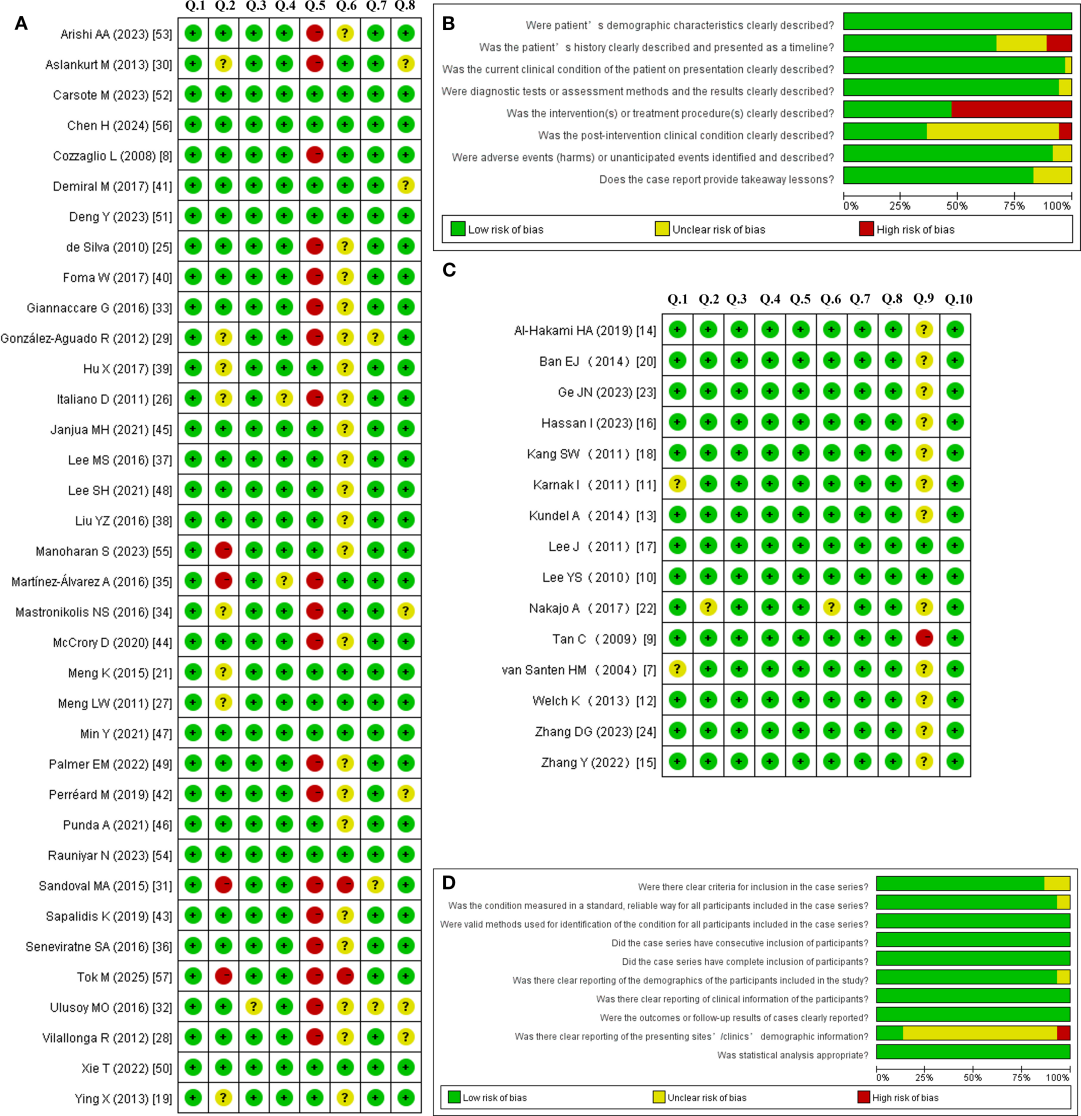
Risk of bias summary and graph for the case reports **(A, B)** and case series **(C, D)** using the Cochrane risk of bias tool.

According to the JBI quality assessment criteria for case reports, only six of the 36 included case reports met all eight criteria. For the majority of case reports, the treatment modalities implemented after the onset of Horner’s syndrome were not recorded; it is also possible that observation alone constituted the treatment strategy. Based on the JBI criteria for case series, only two of the 14 included case series met all ten criteria. Nevertheless, all studies fundamentally met the inclusion criteria and were deemed methodologically suitable for descriptive analysis. **Figure 3** presents the risk of bias assessment for each included study using the Cochrane risk of bias tool.

### 3.1 Incidence of HS

According to **Table 1**, the incidence of HS following TS is 0.25% (95% CI: 0.16%, 0.35%). Among these cases, the incidence for open surgery was 0.41% (95% CI: 0.22%, 0.61%), for endoscopic surgery was 0.15% (95% CI: 0.01%, 0.15%). All the children recorded in the retrieved literature underwent open surgery. The incidence rate among children in open surgery, at 1.84% (95% CI: 0.52%, 4.06%), is higher than that among adults, which is 0.22% (95% CI: 0.14%, 0.34%).

Among the 40 cases, the conventional approach was used in 77.5% (31/40) of cases, while endoscopic surgery—comprising 9 cases of ETS, 1 case of RAET, and 3 cases of VAT—accounted for 22.5% (9/40). Malignant cases constituted 67.5% (27/40) of the total, with benign cases representing the remaining 32.5% (13/40). Surgical procedures included unilateral thyroidectomy (UT), TT, or subtotal thyroidectomy (ST) in 40% (16/40) of patients. Additionally, combined CLND was performed in 32.5% (13/40) of cases, and combined LLND in another 27.5% (11/40) (**Table 3**).

**Table 3:** Summary of surgical approaches for 40 cases.

Approach	Histopathology		Extent of operation		
	Malignancy	Benign	UT, TT or ST	Combined CLND	Combined LLND
Open surgery	18	13	15	6	10
Endoscopic surgery	9	0	1	7	1

UT, unilateral thyroidectomy; TT, total thyroidectomy; ST, subtotal thyroidectomy; CLND, central lymph node dissection; LLND, lateral lymph node dissection.

### 3.2 Time of symptom onset

The onset time of HS was recorded for 38 cases. Among these, HS occurred within 3 days postoperatively in 84.2% (32/38) of cases, including 11 cases on the day of surgery, 6 cases on POD 1, 8 cases on POD 2, and 7 cases on POD 3. Additionally, HS was observed in 1 case on POD 4, 3 cases on POD 7, and 1 case on POD 13, with the longest onset time recorded on POD 27.

### 3.3 Symptoms of HS

Detailed clinical symptoms were documented for all 40 cases. Ptosis was present in 100% (40/40) of patients, miosis in 92.5% (37/40), anhidrosis in 32.5% (13/40), enophthalmos in 20% (8/40), decreased vision in 7.5% (3/40), and heterochromia in 2.5% (1/40). Additionally, 2.5% (1/40) of patients reported symptoms including dry eye, conjunctival irritation, and alternating nasal congestion. Among other recorded complications, five patients developed hoarseness, which was subsequently confirmed by laryngoscopy to be due to ipsilateral vocal cord paralysis; one patient experienced ocular and facial pain; and one patient presented with chylous leakage. No complications such as hematoma or infection were recorded.

### 3.4 Recovery outcome

The recovery outcomes of 36 cases of HS were documented. Complete resolution was achieved in 38.9% (14/36) of cases, with recovery times ranging from 4 days to 11 months. Incomplete resolution was observed in 38.9% (14/36) of cases during the follow-up period (maximum duration: 4 years). No improvement was noted in 22.2% (8/36) of cases (maximum follow-up: 2 years). Among the 14 patients who received immediate pharmacological treatment (steroids and/or neurotrophic therapy) upon HS diagnosis, all exhibited symptom improvement during follow-up, with complete resolution achieved in 7 cases. One case developed a lymphatic cyst due to chylous leakage, and after repeated needle aspiration and drainage, the symptoms completely resolved.

## 4 Discussion

HS refers to a group of clinical syndromes characterized by damage to the CSC (a nerve bundle located on the OSP), leading to nerve paralysis. Common etiologies include inflammation, trauma, surgical procedures, tumors, and thrombus formation. As a rare complication of TS, HS had an incidence of 0.25% in this study. Specifically, the incidence was higher in open - surgery cases compared to endoscopic - surgery cases (0.41% vs. 0.15%).

The mechanisms underlying the development of HS after TS mainly involve direct injury to the CSC, which is attributed to anatomical factors (57). Based on the reviewed literature, the mechanisms of injury can be summarized as follows: 1. Physical damage to the CSC; 2. Ischemia and edema of the CSC resulting from excessive traction and dissection of the carotid sheath; 3. Local compression of the CSC due to the elastic recoil of the carotid sheath; 4. Injury to the middle cervical ganglion (MCG) or its supplying blood vessels during manipulation of the inferior thyroid artery; 5. Excessive traction causing injury to the superior cervical ganglion (SCG) during manipulation of the superior thyroid artery; 6. Injury to the communicating branches between the CSC and the recurrent laryngeal nerve; 7. Thermal injury from energy devices; 8. Compression or infection may occur resulting from postoperative fluid accumulation or hematoma. The occurrence of HS immediately after surgery is related to direct CSC damage, such as excessive traction, thermal injury, or direct transection, while the delayed onset of HS may be due to tissue edema, inflammatory reactions, hematoma compression, or delayed nerve injury. In the case we presented, the patient developed Horner syndrome on POD 3, and the symptoms had not completely resolved even after 2 years. We suspect that this is related to the heat from the ultrasonic scalpel being conducted to the CSC, resulting in irreversible damage.

Understanding the anatomical basis of the OSP and CSC can further deepen our comprehension of HS. The OSP, composed of a pathway with three types of neurons, originates from the central nervous system, traverses the CSC, and ultimately reaches the eye (58). The second - order neurons, within which the CSC is located, are closely associated with iatrogenic injuries. The CSC is located posterior to the carotid sheath, anterior to the longus muscles, inferior to the prevertebral fascia, and is linked to the superior, middle, and inferior cervical ganglia. Excessive traction, dissection, extensive surgical procedures, or complex thyroid surgeries can cause CSC damage, which increases HS risk (59).

In the studied cases, malignant surgeries outweighed benign ones (67.5% vs. 32.5%), with 60% (24/40) undergoing lymph node dissection. HS incidence was high after specific surgeries, such as 27.3% (3/11) in medullary thyroid carcinoma cases involving TT+ CLND+LLND (5). Another study, encompassing 45 patients who underwent LLND, the incidence of HS was 4.4% (2/45) (11). When the tumor is intimately associated with the carotid sheath, surgical dissection of this structure significantly contributes to CSC damage (27, 30). Close adhesions between metastatic lymph nodes and the carotid sheath can lead to misidentification and inadvertent removal of ganglia. Therefore, it is evident that extensive cervical lymph node metastasis with subsequent LLCD is a significant factor contributing to postoperative HS. Additionally, McCrory (43) reported a case in which a cervical ganglioneuroma was mistakenly identified as an ectopic parathyroid adenoma and subsequently removed, ultimately resulting in HS.

Communicating branches between the recurrent laryngeal nerve and the CSC are present in some individuals (60). During the dissection and identification process of the recurrent laryngeal nerve, there exists a potential risk of damaging these communicating branches. In this study, five patients developed HS along with ipsilateral vocal fold paralysis, which underscores the close relationship between these two conditions.

The MCG, representing the smallest ganglion within the CSC, is located in close proximity to the inferior thyroid artery and may receive its blood supply from this artery (57). Consequently, during the dissection of the inferior thyroid artery, there exists a risk of directly damaging the MCG. Moreover, ligation of these vessels may result in ischemic injury to the MCG, thereby triggering the onset of HS (8).

The parapharyngeal space is another anatomical site that warrants particular attention. At the C1 - C4 levels, the SCG, which constitutes the largest ganglion within the CSC, is situated posterior to the carotid sheath and is anatomically contiguous with the parapharyngeal space. Excision of parapharyngeal metastatic lymph nodes (14), ectopic thyroid tissue (39), and excessive dissection of the upper pole during ETS (55) can result in HS, and all these cases may be associated with SCG injury.

A study indicates that although the incidence of HS after endoscopic surgery is lower than that after open TS, the incidence of HS remains relatively high during the initial phase of implementing endoscopic surgery (61). However, the results of this review cannot effectively support this conclusion. When performing endoscopic surgery to manage the superior pole of the thyroid, it is often necessary to retract the carotid sheath within the confined surgical space to adequately expose the operative field (55). Using a retractor to retract the carotid sheath carries the risk of exposing or injuring the SCG.

Energy - based devices, especially ultrasonic instruments that provide the dual benefits of cutting and coagulation, have been extensively employed in a variety of surgical procedures and play a pivotal role in endoscopic surgery. During surgical operations, thermal injury resulting from ultrasonic instruments represents another potential etiological factor for nerve damage, particularly in patients with anatomical variations of the CSC. In a rat model study, Carlander (62) reported that the local thermal energy effects generated by ultrasonic instruments can induce neurological dysfunction, and the severity of nerve damage is contingent upon the duration of thermal exposure. Furthermore, other studies have demonstrated that the incidence of temporary recurrent laryngeal nerve paralysis is elevated following the use of ultrasonic instruments compared to traditional techniques (63). Similarly, thermal ablation employs thermal energy to induce coagulative necrosis of tumor tissues, which may potentially cause thermal injury to the CSC (64).

Carsote (51) documented a case of chylous leakage following TS. Insufficient drainage resulted in the development of a lymphatic cyst, which subsequently exerted compression on the CSC and led to HS. After undergoing repeated needle aspiration for fluid drainage, the patient’s symptoms resolved completely. This indicates that postoperative fluid accumulation, lymphatic cysts, or hematomas that compress the CSC can also serve as a causative factor for HS.

Although the classical manifestations of HS encompass ptosis, miosis, and anhidrosis, the findings from the aforementioned literature indicate that ptosis and miosis are more prevalent than anhidrosis. Ptosis arises from the loss of sympathetic tone in Müller’s muscle (which is capable of elevating the upper eyelid by approximately 1–2 millimeters), leading to a narrowed palpebral fissure (65). There exists a muscle with a comparable function in the lower eyelid; consequently, in HS, the lower eyelid may be slightly elevated (occasionally termed ‘upside - down ptosis’) (66). It is noteworthy that, despite ptosis being mentioned in all these records, one study revealed that 12% of patients did not manifest symptoms of ptosis (67). When the symptoms are mild, the lower eyelid may also close more tightly. This condition imparts a sunken appearance to the eyes even in the absence of actual enophthalmos; true enophthalmos is relatively rare (68). The sympathetic cholinergic fibers within the OSP govern the function of facial sweat glands, and disruption of this pathway can result in anhidrosis. In specific cases, OSP dysfunction can induce facial flushing, conjunctival congestion, lacrimation, and nasal congestion due to vasodilation in the innervated regions (51).

When HS manifests subsequent to TS, a differential diagnosis should be carried out in collaboration with departments including neurology and ophthalmology. This is to rule out potential etiologies, such as intracranial lesions, cervical spinal cord lesions, cervicothoracic tumors, infections, immunologic diseases, or carotid artery lesions, prior to considering HS as a complication of TS.

The apraclonidine test represents the simplest and most reliable diagnostic modality for HS in adults, as it induces mydriasis of the affected pupil owing to the upregulation of α - 1 postsynaptic receptors (69). A potential limitation is that the test is contingent upon the presence of hypersensitivity, suggesting that OSP dysfunction must have persisted for a sufficient period to facilitate the upregulation of postsynaptic receptors (70). Consequently, the test exhibits limited diagnostic utility in the acute phase of HS but is generally deemed reliable two weeks after the onset of symptoms. Apraclonidine eyedrops are also capable of elevating the affected eyelid, offering temporary cosmetic amelioration for ptosis (71). Cocaine eyedrops serve as the preferred diagnostic agent for the diagnosis of HS (72). They inhibit the presynaptic uptake of norepinephrine, thereby elevating its concentration in the synaptic cleft and resulting in pupil dilation (73). A positive test outcome is indicative of HS when the mydriasis on the suspected side is significantly less than that on the healthy side (74).

HS typically does not result in functional impairment; however, alterations in appearance may lead to psychological distress. Short - term administration of steroids in conjunction with neurotrophic therapy can facilitate neuronal repair and aid in the alleviation of these symptoms. In the present study, 7.5% (3/40) of the cases presented with decreased visual acuity, and 2.5% (1/40) of the patients developed heterochromia. These findings suggest that patients with concurrent HS following TS necessitate close monitoring of ocular functional changes to enable timely intervention and prevent disease progression. Additionally, the results demonstrated that complete recovery occurred in all cases within one year, with the likelihood of complete recovery significantly diminishing after this time frame. It is noteworthy that, among the retrieved literature, no definition has been provided for the term “incomplete resolved.” Based on our case, during the two - year follow - up period, the patient’s ptosis and anhidrosis demonstrated improvement, whereas the degree of miosis remained unchanged. Another concern is that, for the group of patients categorized as ‘no improvement’ and ‘incomplete resolved,’ the assessment of HS outcomes may have been biased due to short follow-up durations or loss to follow-up. Therefore, we contend that the statistically reported HS outcomes in the article do not accurately reflect the true prognosis of this complication, as they omit the process of long-term follow-up and management.

Additionally, although IONM was employed in our case to ensure the normal function of the recurrent laryngeal nerve and the vagus nerve, HS still developed. This indicates that IONM may not be able to prevent CSC injury, which is consistent with previous reports (75). IONM primarily focuses on monitoring motor nerves, such as the recurrent laryngeal nerve and the vagus nerve; however, it does not provide feedback on autonomic nerve fibers, like those that constitute the CSC (76). Therefore, relying solely on IONM without direct visualization or anatomical knowledge of the CSC may give a false sense of security regarding nerve preservation during thyroid surgery. Surgeons should be aware of these limitations and integrate direct visualization and anatomical knowledge into their surgical techniques to minimize the risk of CSC injury.

This study has several limitations. The included literature is limited in both quality and quantity, with substantial heterogeneity present in research methodologies. This increases the difficulty of data extraction and comprehensive analysis. The studies are predominantly observational in nature, which makes it difficult to establish a definitive causal relationship between TS and HS, and mechanistic investigations remain speculative. There is insufficient data on pediatric patients and specific surgical techniques, impeding the assessment of their impact on specific patient subgroups and surgical modalities. Inconsistent follow-up durations and outcome assessment criteria reduce the comparability of results and the thorough evaluation of the long - term effects of HS.

Although this study conducted a systematic review following the PRISMA guidelines (77), its methodological rigor still did not fully meet the expected level of the Cochrane/Campbell standards. Future research endeavors ought to utilize multicenter prospective cohorts or randomized controlled trials (RCTs) to establish causality. Animal models, in conjunction with intraoperative neuromonitoring, can offer insights into the underlying mechanisms. Particular attention should be directed towards pediatric populations and minimally invasive surgical techniques, accompanied by long - term follow - up (≥5 years) to evaluate chronic outcomes. The establishment of a global registry and the development of multidisciplinary guidelines would further enhance the management of HS.

## 5 Conclusion

HS is a clinical manifestation that describes the impairment of the CSC, representing a rare and non-fatal complication following TS, which arises from multiple injury mechanisms. Although HS typically does not affect ocular function, it causes significant cosmetic and psychological distress to patients, particularly after endoscopic surgeries that are intended to enhance appearance. In rare cases, decreased vision and heterochromia may also occur. For acute symptoms of CSC injury following surgery, diagnosis can be made using the apraclonidine test and cocaine eyedrops. Once HS is diagnosed, short-term steroid and neurotrophic treatments can be administered. If complete recovery is not achieved within a year, the likelihood of complete recovery decreases significantly thereafter. Therefore, patients should be fully informed of this risk prior to surgery, and the area surrounding the CSC should be carefully assessed and operated on with precision to avoid damaging it. However, currently, substantial disparities exist in the evidence level and heterogeneity among the published literature. In the future, it is imperative to establish a comprehensive recording system to analyze the etiology and prognosis of such rare complications.

## Data Availability

The original contributions presented in the study are included in the article/supplementary material. Further inquiries can be directed to the corresponding authors.
